# Fat and vitamin intakes during pregnancy have stronger relations with a pro-inflammatory maternal microbiota than does carbohydrate intake

**DOI:** 10.1186/s40168-016-0200-3

**Published:** 2016-10-19

**Authors:** Siddhartha Mandal, Keith M. Godfrey, Daniel McDonald, Will V. Treuren, Jørgen V. Bjørnholt, Tore Midtvedt, Birgitte Moen, Knut Rudi, Rob Knight, Anne Lise Brantsæter, Shyamal D. Peddada, Merete Eggesbø

**Affiliations:** 1 Department of Environmental Exposure and Epidemiology, Norwegian Institute of Public Health, Oslo, Norway; 2 Present address: Public Health Foundation of India, Gurgaon, India; 3 MRC Lifecourse Epidemiology Unit and NIHR Southampton Biomedical Research Centre, University of Southampton and University Hospital Southampton NHS Foundation Trust, Southampton, UK; 4 Department of Pediatrics at the University of California San Diego, San Diego, CA USA; 5 Department of Microbiology and Immunology, Stanford University, Stanford, CA USA; 6 Microbiological Department Oslo University Hospital, Oslo, Norway; 7 Institute for Clinical Medicine, University of Oslo, Oslo, Norway; 8 Department of Microbiology, Tumor and Cell biology (MTC), Karolinska Institute, Stockholm, Sweden; 9 The Norwegian Institute of Food Fisheries and Aquaculture, Aas, Norway; 10 Department of Chemistry, Biotechnology and Food Science, Norwegian Institute of Life Sciences, Aas, Norway; 11 Department of Computer Science, UC San Diego, San Diego, CA USA; 12 Biostatistics and Computational Biology Branch, National Institute for Environmental Health Sciences, Durham, NC USA

**Keywords:** Compositional shifts, Vitamin D, Mono-unsaturated fat, *Proteobacteria*

## Abstract

**Background:**

Although diet is known to have a major modulatory influence on gut microbiota, knowledge of the specific roles of particular vitamins, minerals, and other nutrients is limited.

Modulation of the composition of the microbiota in pregnant women is especially important as maternal microbes are transferred during delivery and initiate the colonization process in the infant. We studied the associations between intake of specific dietary nutrients during pregnancy and gut microbiota composition.

**Methods:**

Utilizing the Norwegian NoMIC cohort, we examined the relations between intakes of 28 dietary macro- and micronutrients during pregnancy, derived from food frequency questionnaires administered to 60 women in the second trimester, and observed taxonomic differences in their gut microbiota four days after delivery (assessed through Illumina 16S rRNA amplicon analysis).

**Results:**

Higher dietary intakes of fat-soluble vitamins, especially vitamin D, were associated with reduced microbial alpha diversity (*p* value <0.001). Furthermore, using recently developed statistical methodology, we discovered that the variations in fat-soluble vitamins, saturated and mono-unsaturated fat, and cholesterol intake, were associated with changes in phyla composition. Specifically, vitamin D, mono-unsaturated fat, cholesterol, and retinol were associated with relative increases in *Proteobacteria*, which is a phylum known to encompass multiple pathogens and to have pro-inflammatory properties*.* In contrast, saturated fat, vitamin E, and protein were associated with relative decreases in *Proteobacteria*.

**Conclusions:**

The results in this article indicate that fats and fat-soluble vitamins are among the most potent dietary modulators of gut microbiota in mothers. The shifts in microbiota due to diet need to be further studied alongside gut microbiota changes during pregnancy to better understand the impact on infant gut microbiota.

**Electronic supplementary material:**

The online version of this article (doi:10.1186/s40168-016-0200-3) contains supplementary material, which is available to authorized users.

## Background

Among all factors which affect human gut microbiota (GM), dietary intake is one of the most important and easily manipulated [1]. Dietary nutrients modulate gut microbiota which in turn affects human health and diseases [2, 3]. Scott et al. (2013) provided an extensive review of the effects of modulating dietary components on gut microbiota. Research on germ-free mice has demonstrated host-microbial interactions whereby microbes develop symbiotic relationships, deriving energy from nutrients that are undigested by the host [4, 5].

Most research examining the influence of diet on gut microbes has focused on combinations of resistant starch, polysaccharides, and fats. Several authors have studied the effect of specialized diets on overweight subjects [2, 6–8]. Typical findings in these studies revolve around a decrease in carbohydrate intake causing a decrease in butyrate and decrease in butyrate producing bacteria, such as *Roseburia* and *Eubacterium*. However, most of these studies are based on specific dietary regimes and small sample sizes.

There is an extensive literature studying the effect of vitamin deficiency (especially vitamins A and D) on immune responses [9, 10] and other health effects such as macular degeneration and musculoskeletal health. It is plausible that some of the effects of vitamins are mediated through an effect on GM composition, yet there is limited literature on the associations between vitamins or minerals and gut microbiota. Antimicrobial effects of vitamin D have been reported: Vitamin D boosts innate immunity by facilitating production of anti-microbial peptides and cytokines [11]. Further, vitamin D has also been shown to affect microbes such as *Mycobacterium tuberculosis* and hence slowing the progression to tuberculosis [12]. Gut bacteria can also affect vitamin concentration. For example, LeBlanc et al. (2013) reviewed some possible mechanisms for de novo synthesis of vitamins by lactic acid bacteria [13].

Microbiota is remodeled at several sites in the body during pregnancy, likely in preparation for childbirth. In a longitudinal study, Romero et al. (2014) demonstrated that the vaginal microbial composition of a pregnant woman differs from a non-pregnant woman, including increased abundance of *Lactobacillus* and decreased abundance of phylotypes associated with bacterial vaginosis [14]. Collado and Isolauri (2008) reported differences in gut microbial composition [15], specifically *Bacteroides*, *Clostridium*, and *Staphylococcus*, between overweight and normal-weight women during pregnancy. Koren et al. (2013) studied metabolic changes and remodeling of the gut microbiome between the first and third trimesters of pregnancy [16]. During this period, they reported a dramatic change in gut microbiota composition, including an increase in *Actinobacteria* and *Proteobacteria* and a reduction in species richness.

Thus, both pregnancy itself and dietary quality and micronutrient content may affect gut microbial composition. The composition of the microbiota in a pregnant woman at delivery likely affects the gut microbiome of the newborn, because maternal microbes are transferred during passage through the birth canal and initiate the colonization process in the infant [17, 18]. Young children have substantially similar gut microbiota to their own mothers than to unrelated individuals [19]. Increased knowledge of how foods and nutrients can modulate gut composition in the pregnant women is thus of importance, not only for the mother but also for the unborn child.

In this article, we analyzed the relationships between dietary intake of 28 nutrients assessed by a food frequency questionnaire administered during pregnancy and gut microbiota in close conjunction with delivery. We utilized the NoMIC cohort, a cohort which oversampled preterm deliveries, and studied the 60 mothers out of 550 who also had available prospective dietary data collected. In addition to the intake of carbohydrates, fats, and protein, we also studied individual micronutrients. Using recently developed statistical methodologies [20], we analyzed dietary intake in relation to both microbial diversity and microbial composition in an attempt to understand the ways in which dietary components modulate the gut microbiota during pregnancy.

## Results

### Characteristics of the variables of interest

**Fig. 1:**
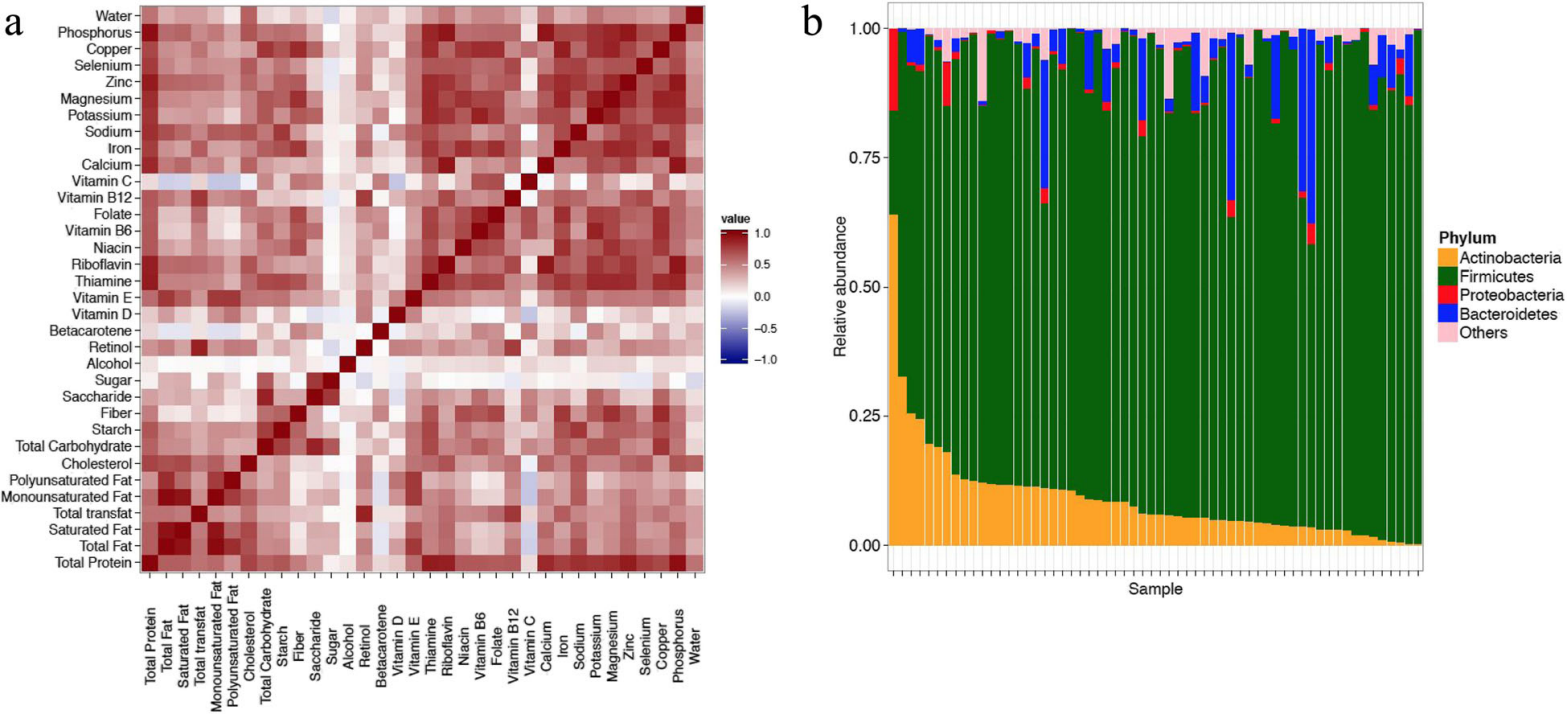
Correlation of dietary components and microbial compositions for subjects. **a** Heatmap showing correlations among the 34 dietary components using Pearson’s correlation coefficient between the standardized dietary components. **b** Relative abundances of major phyla in the 60 subjects, arranged according to decreasing relative abundance of *Actinobacteria*

Table 1 shows descriptive statistics for the dietary components and other variables included in subsequent analyses. In Fig. 1a, we present the standardized intakes of the dietary components and the correlations between these components. The figure shows that intakes of most dietary components (except alcohol and sugar) were positively correlated with each other. In Fig. 1b, individual specific phyla level microbial composition is presented in terms of relative abundances of *Actinobacteria*, *Proteobacteria*, *Firmicutes*, *Bacteroidetes*, and *Other*, where *Other* comprise all remaining phyla.

**Table 1:** Characteristics of 60 women participating in the NoMIC study according to demographic variables

Variable	Mean	SD
Gestational age (in days)	264.3	27.2
Pre-pregnancy BMI	22.9	3.5
Pre-pregnancy weight (in kg)	64.7	10.6
Weight at delivery (in kg)	78.5	10.9
	*n*	Percentage
Preterm delivery		
No	39	65.0
Yes	21	35.0
Mode of delivery		
Vaginal	43	72.0
C-section	17	28.0
Maternal education		
Less than 12 years	5	8.0
Equal to 12 years	9	15.0
More than 12 years	46	77.0
Maternal smoking		
No	45	75.0
Yes	15	25.0
Maternal antibiotics		
Anytime during pregnancy	17	28.0
Month before birth	4	6.7
Supplement used (third trimester)		
Vitamin	18	30.0
Cod liver oil	17	28.0
Omega 3	24	40.0

### Association of dietary nutrients with gut microbiota

**Fig. 2:**
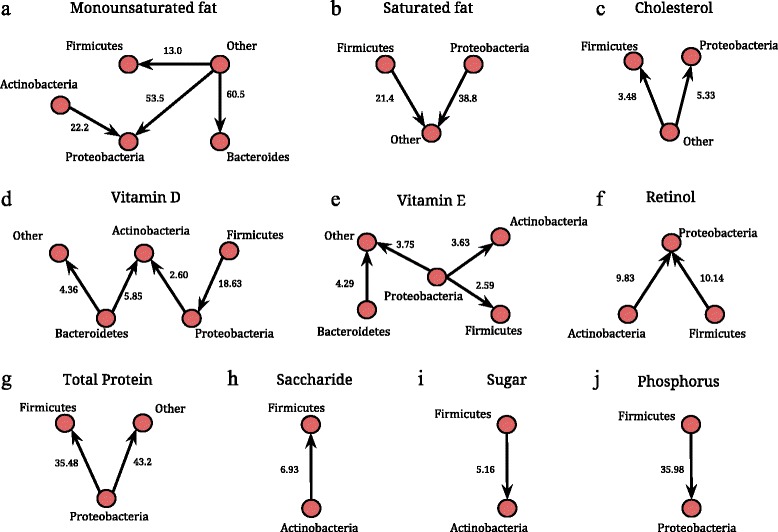
Associations of increase in specific dietary nutrients with major microbial phyla, *Actinobacteria*, *Proteobacteria*, *Firmicutes*, *Bacteroidetes*, and *Other* (comprised of all other phyla) based on a multiple linear regression model. Panels **a**-**j** show the significant shifts in microbial composition against specific nutrients using networks. In each network, a *node* represents a phylum and a directed *arrow* from Phyla 1 to Phyla 2 represents a statistically significant increase. The value (*x*) on each edge represents a x-fold increase in Phyla 2 compared to Phyla 1, with each unit standard deviation (SD) increase in the corresponding dietary variable. The value (*x*) is calculated as exp(*β*), where *β* is the regression coefficient corresponding to the linear regression of the ratio Phyla 2/Phyla 1 on the dietary variables. For example, there is a 3.63 times increase in *Actinobacteria* compared to *Proteobacteria* for 1 SD increase in vitamin E intake

**Fig. 3:**
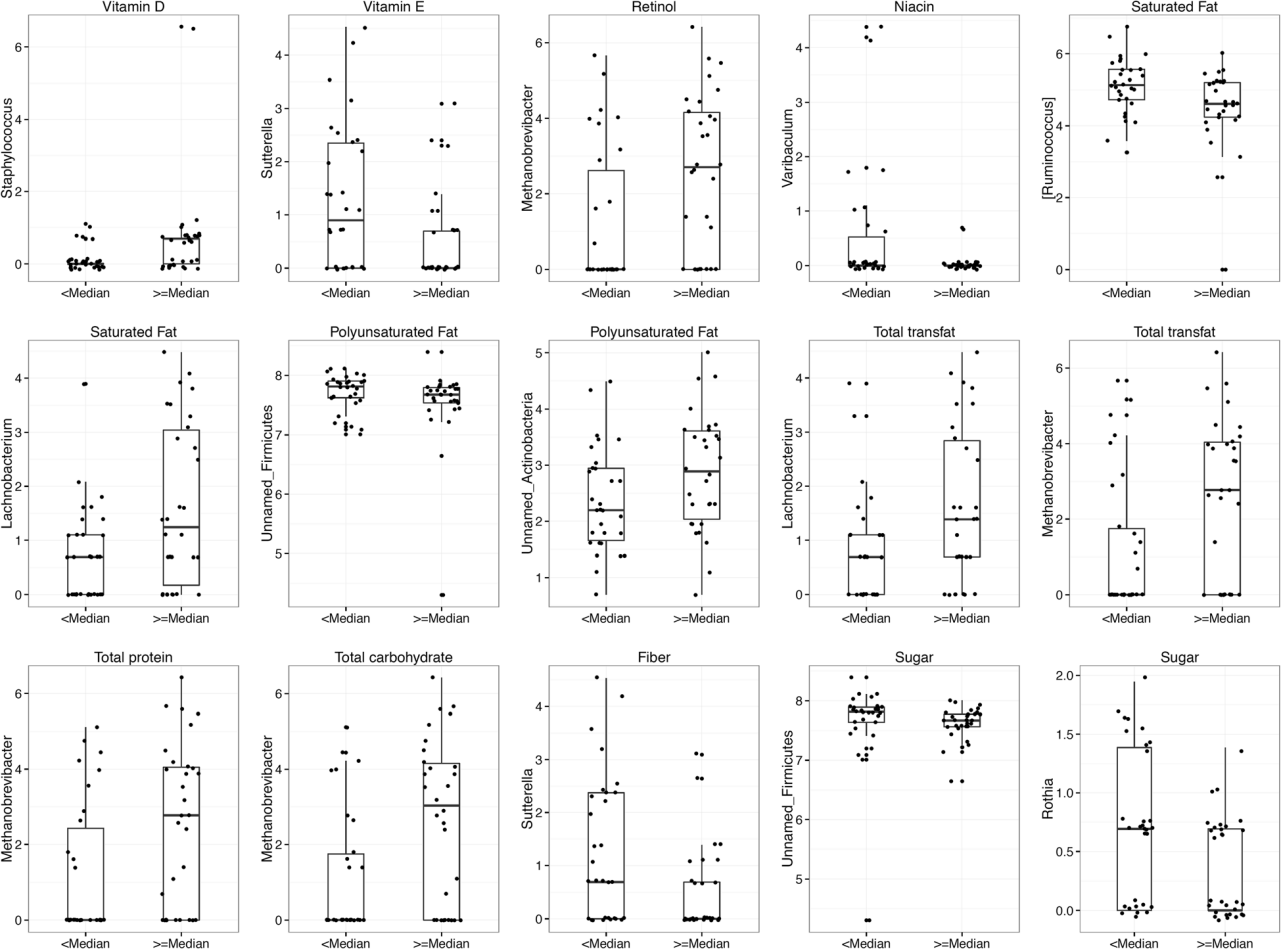
Boxplots to describe log OTU abundances of differentially abundant genera identified by a compositional analysis against the dietary variables, categorized as below and above the median intake of the corresponding nutrient. Genera abundance is obtained from OTU table summarized at genus level and testing procedure based on log-ratio analysis is described in Additional file 1

- Microbial diversity
  In order to explore the associations between nutrient intakes and diversity in gut microbiota, we employed a variable selection using Bayesian model averaging (Additional file 1: Figure S1). For both whole tree phylogenetic and Shannon’s diversity, vitamin D showed the strongest statistical associations. Other variables associated with phylogenetic diversity were other fat-soluble vitamins (retinol and vitamin E), cholesterol, and trans-fat. However, the inclusion probabilities for all variables other than vitamin D were small. We then used linear regressions, adjusting for maternal pre-pregnancy BMI and parity, to quantify the effects of these variables. For whole tree phylogenetic diversity, significant inverse associations were observed for vitamin D, retinol, and cholesterol (% change in diversity per unit increase of the vitamin; −7.8 %, *p* = 0.001, −5.6 %, *p* = 0.031, −5.3 %, *p* = 0.043, respectively) (Additional file 1: Table S1). In contrast, for Shannon’s diversity, only vitamin D was significantly (and negatively) associated (−5.1 % change in diversity per unit increase in vitamin D intake, *p* < 0.001). Measures of beta diversity such as weighted and unweighted UniFrac distances did not show any clustering according to the dietary components (data not shown).
- Microbial composition
  We explored the associations of 28 specific nutrients (Table 2) in relation to microbial phyla composition (shown in Fig. 2) and on microbial genera (shown in Fig. 3), and these findings are summarized below by nutrient group. Detailed results containing the estimates of the regression coefficients, *p* values, and *R* ^2^ for the models are provided in the Additional file 1: Table S2.

**Table 2:** Median and inter-quartile ranges for the dietary components in 60 women in the analysis

Dietary component	Units	Median	IQR	Minimum	5th percentile	95th percentile	Maximum
Energy	kJ	9693.11	2944.616	4446.9238	6444.502	12274.603	15628.0185
Total protein	g	88.29	24.41	47.315	60.077	117.199	138.4564
Total fat	g	72.00	29.17	42.6707	49.028	119.742	163.0225
Saturated fat	g	28.28	10.57	16.2139	18.763	49.097	70.2995
Total trans-fat	g	2.22	1.46	0.7147	1.031	4.547	8.7354
Monounsaturated fat	g	22.50	8.17	13.061	15.343	37.794	47.3162
Polyunsaturated fat	g	13.50	5.62	7.1054	8.904	27.798	32.4678
Cholesterol	g	0.22	0.10	119	153.8	411.7	473
Total carbohydrate	g	308.97	77.66	121.9611	195.504	404.833	536.0477
Starch	g	143.64	51.91	60.1423	91.217	215.446	225.462
Fiber	g	31.88	12.99	9.7115	19.917	47.254	55.1066
Saccharide	g	145.18	65.84	35.7406	89.833	214.339	294.8139
Sugar	g	53.40	38.58	13.1777	18.267	123.172	213.0867
Alcohol	g	0.00	0.00	0	0	0.019	2.2635
Retinol	μg	822.50	746.00	136	190.1	2373.25	4575
Betacarotene	μg	1894.00	1661.00	607	1021.9	5271.4	9934
Vitamin D	μg	3.13	2.34	0.5386	0.856	6.517	34.1235
Vitamin E	mg	9.50	4.00	4	5	16	17
Thiamine	mg	1.58	0.51	0.739	1.039	2.047	2.5115
Riboflavin	mg	1.89	0.92	0.6309	1.065	2.808	4.2915
Niacin	mg	19.97	5.63	11.828	14.558	26.228	28.8022
Vitamin B6	mg	1.54	0.56	0.8507	1.051	2.07	2.6655
Folate	μg	266.50	93.75	130	171.85	409.5	537
Vitamin B12	μg	5.66	4.22	2.4648	2.863	12.607	15.3611
Vitamin C	mg	158.00	107.00	39	52.6	287.8	406
Calcium	mg	914.00	464.75	356	461.7	1617.6	2249
Iron	mg	11.80	3.12	4.9057	7.296	15.636	20.3218
Sodium	mg	3159.50	809.50	1832	2094.7	4442.2	4904
Potassium	mg	3992.00	1429.25	2004	2725.15	5319.05	7468
Magnesium	mg	404.00	126.25	170	267	511	689
Zinc	mg	11.82	3.29	5.7411	7.295	14.78	20.1115
Selenium	μg	53.00	13.50	28	33.85	73.05	88
Copper	mg	1.46	0.42	0.7122	0.98	1.792	2.2134
Phosphorus	mg	1733.00	530.00	733	1115.3	2303.85	3073
Water		3144.00	1399.00	1427	1721.55	4423.55	7292

### Fat-soluble vitamins (vitamins D, E, and retinol)

We detect significant associations between the three fat-soluble vitamins (vitamin D, E, and retinol) and microbial composition at the phylum level (expressed through pairwise ratios of microbial phyla *Phylum* 1/*Phylum* 2). Vitamin D was associated with increases in *Actinobacteria*/*Proteobacteria, Actinobacteria*/*Bacteroidetes*, *Proteobacteria*/*Firmicutes*, and *Other*/*Bacteroidetes* (Fig. 2d)*.* Higher intake of vitamin E was associated with a decrease in *Proteobacteria* relative to *Actinobacteria*, *Firmicutes* and *Other*, and increase in from *Other*/*Bacteroidetes* (Fig. 2e), while increase in retinol was related to the increase in the ratio of *Proteobacteria* relative to *Actinobacteria* and *Firmicutes* (Fig. 2f). At the genus level, we observed the following differentially abundant genera against increasing intake of fat-soluble vitamins; *Staphylococcus* (higher relative abundance) against vitamin D, *Sutterella* (lower) against vitamin E, and *Methanobrevibacter* (higher) against retinol (Fig. 3, top row).

### Fat components

Increased saturated fat intake was related to an increase in the *Other*/*Proteobacteria* as well as *Other*/*Firmicutes* (Fig. 2b)*.* Interestingly, the converse appears to be true with respect to monounsaturated fat, where an increase in the intake of monounsaturated fat resulted in a significant increase in *Firmicutes*, *Proteobacteria*, and *Bacteroidetes* relative to *Other* phyla (Fig. 2a). Furthermore, there was a significant reduction of *Actinobacteria*/*Proteobacteria.* Increased cholesterol intake resulted in a significant increase in *Firmicutes* and *Proteobacteria* relative to *Other* phyla (Fig. 2c)*.* At the genus level, we identify *Ruminococcus* (lower relative abundance) and *Lachnobacterium* (higher) to be differentially abundant against increased saturated fat intake. *Lachnobacterium* and *Methanobrevibacter* were detected (higher relative abundance) against increased intake of trans-fat. We also identified microbes belonging to *Firmicutes* and *Actinobacteria* (that were not identified at the genera level) to be decreased and increased, respectively, against increased intake of polyunsaturated fat (Fig. 3, top and middle row).

### Protein, carbohydrate, and minerals

Increased intake of protein was related to a significant decrease of *Proteobacteria*/*Firmicutes* and *Proteobacteria*/*Other* (Fig. 2g). Among carbohydrates, we found that an increased intake of saccharide significantly reduced the ratio *Actinobacteria*/*Firmicutes* (Fig. 2h) but an opposite relationship was seen with an increased intake of sugar (Fig. 2i). Finally, among all minerals, increased phosphorus levels resulted in a significant increase in *Proteobacteria* relative to *Firmicutes* (Fig. 2j). *Methanobrevibacter* was the only genus detected as differentially abundant against an increased intake of total protein and carbohydrates. Against increased fiber intake, *Sutterella* was differentially abundant while *Rothia* and unclassified *Firmicutes* were detected against increased sugar intake (Fig. 3, bottom row).

### Sensitivity analysis

Since the NoMIC cohort involved an oversampling of preterm mothers, it is not representative of the general Norwegian population. In addition, maternal pre-pregnancy BMI is a potential confounder of the diet-microbe associations. Hence, we carried out the above analysis while adjusting for gestational age and maternal BMI and provide the results in the Additional file 1: Table S3. There were few changes in the effect sizes with the significance pattern remaining unchanged for most associations. Overall gestational age was a significant factor only for the ratios of *Actinobacteria* relative to *Firmicutes* and *Proteobacteria*, with a decrease in *Actinobacteria*. For the diet-related associations, the effects of saccharide and sugar were only marginally significant on *Actinobacteria* relative to *Firmicutes*, although the effect sizes were similar. In addition, mono-unsaturated fat, thiamine, and vitamin C were significantly associated with *Actinobacteria*/*Firmicutes*. The associations between total protein intake with *Proteobacteria*/*Others* and vitamin D with *Bacteroidetes*/*Others* are not statistically significant after adjusting for gestational age. As an additional sensitivity analysis, we provide the dietary association results within the strata of term deliveries (Additional file 1: Table S4) and observed that the effect sizes are consistent with the earlier analysis.

We also performed sensitivity analysis for the associations of fat-soluble vitamins and microbial composition in mothers who did not use vitamin D, vitamin E, retinol (vitamin A) supplements, eg., restricting solely to dietary intakes and achieved similar results for most associations (Additional file 1: Table S5).

## Discussion

In this article, we studied the relationship between intake of dietary components during pregnancy and maternal gut microbiota assessed four days after delivery. Several of our findings are important and novel due to lack of literature on diet-microbiota associations during the crucial period of pregnancy and the detected associations with multiple nutrients other than the commonly studied carbohydrates, fat, and protein components. We observed that dietary intakes of fat-soluble vitamins, as well as variations in saturated and mono-unsaturated fat intake, were associated with significant changes in phyla composition. The largest number of significant associations was observed with *Proteobacteria*, a phylum known to encompass multiple pathogens and to have pro-inflammatory properties [21]. Specifically, mono-unsaturated fat, vitamin D, and retinol were associated with shifts towards *Proteobacteria*.

Using Bayesian variable selection, one of our other main findings was that higher vitamin D intake was specifically associated with decreased alpha diversity. Low microbial diversity is often associated with diseases such as inflammatory bowel disease [22, 23], obesity [24], autism [25], allergy [26], and asthma [27, 28]. This finding is thus in contrast to studies proposing high vitamin D intake as protective against some of the same disorders, for instance asthma [29]. However, there is support in the literature for high vitamin D intake also being associated with adverse health effects [30]. Increased intakes of vitamin D were also associated with increased levels of *Actinobacteria* and *Proteobacteria* at the phylum level, which are known to encompass multiple low-pathogens and *Staphylococcus* at the genus level. The associations with vitamin D might perhaps be explained by its anti-microbial properties, whereby higher intake suppresses certain groups of bacteria thereby leading to a relative increase in possibly pathogenic groups. However, more studies are needed to clarify the role between dietary intake of vitamin D and health and also on the role of contaminants that may coexist in citamin D-rich food. Interestingly, vitamin E and retinol had decreasing and increasing effects on *Proteobacteria*, respectively.

Lower vitamin E and fiber intakes were associated with higher levels of *Sutterella*, which has been reported to be higher in abundance in infants with autism and some gastrointestinal disorders [31]. Further research might be warranted to confirm or refute these findings and delineate possible mechanistic role of fat-soluble vitamins in autism spectrum disorders.

Interestingly, saturated and mono-unsaturated fat intakes had opposing associations with the gut microbiota at phyla level. A higher dietary intake of mono-unsaturated fat and cholesterol, linked with adverse effects on human health, was associated with relative increase in the *Proteobacteria* phylum. On the other hand, saturated fat was associated with decreases in *Firmicutes* and *Proteobacteria*. The result suggests that certain types of fat may be associated with a pro-inflammatory gut microbiota, which could be a potential explanation for its adverse effects on human health.

Higher intake of proteins and carbohydrates has been associated with an increase in methanogens, and certain methanogens have been identified as important in removal of excess hydrogen and increasing the yield of energy from nutrients [32]. However, detailed knowledge on the role of these bacteria on human health is still unclear. The lack of associations between phyla composition and fiber and sugar (often reported in studies of dietary regime changes) could be attributed to less variation in these factors in our study sample.

Studies such as Koren et al. (2012) and Yatsunenko et al. (2012) have shown that maternal gut microbiota changes during pregnancy and is associated with gestational age at delivery, with increase in *Proteobacteria* and inflammation in the mucosal surfaces. However, in our study, the associations between dietary intake and maternal gut microbiota were not significantly influenced by gestational age. Higher gestational age was significantly associated with an increase in *Actinobacteria*; however, very few dietary associations were altered in terms of statistical significance after adjustment for gestational age. This indicates that gestational age is not a confounder for the relationship between diet and gut microbiota as it is only tied to one of the factors and not both, e.g., gestation is not associated with maternal diet. Thus, the oversampling of preterm mothers in the cohort did not affect the results. In addition, adjustments for maternal pre-pregnancy BMI provided similar results.

Although this study focused on the gut microbiota, it is reasonable to assume that dietary nutrients may also affect the microbiota in other bodily locations such as the mouth and skin. Furthermore, information regarding microbial metabolites including short-chain fatty acids could further enhance our understanding of the potential mechanism underlying these findings.

### Limitations

The major limitation of this study is its small sample size, yet we observe strong signs of association. Although this study focuses on Norwegian mothers, an influence of dietary vitamin and fat intakes is likely to be generalizable to pregnant women in other settings. Further studies will be required to define if the findings are relevant for the adult population in general.

Our food frequency questionnaires (FFQ) estimates of nutrient intakes are likely to contain inaccuracies. However, the FFQ-based intake estimates were validated against 4-day weighed food diaries and good correlations observed for most nutrients except vitamin E. The estimates of vitamin D intake was in addition validated against analysis of plasma 25-hydroxy-vitamin D concentrations and found to distinguish between high and low vitamin D intake [33]. In this paper, we were interested in the effects of dietary intake over the longer term, for which more accurate dietary intake assessment methodologies are not feasible. Finally, any misclassification of intake is likely to move the estimates towards the null.

Maternal gut microbiota was sampled four days after delivery. Sampling during delivery would have been even more optimal but not feasible in this study. Misclassification in dietary exposure would bias the results towards the null and thus reduce the chances of observing an effect. Short-term dietary exposures would probably have revealed more associations; however, in our case, the dietary information is aggregated over long term and hence we believe that such long-term observed effects are indeed interesting.

From the multivariate models, we observed that the *R* ^2^ values range from 0.62 to 0.37 (0.86 to 0.37 after adjusting for gestational age) and hence other factors such as hormonal- and delivery-related changes could be factors that are associated with maternal gut microbiota composition. If we consider a directed acyclic graph for the association of diet with gut microbiota, hormonal change is not a confounder since it is unlikely to affect the dietary intake during pregnancy. Hence, there is little reason to believe that these would introduce a bias with regard to previous intake of vitamins and fat.

Confounding by other factors that may affect dietary intake cannot be excluded. However, our results were surprisingly unaffected when adjusting for the most obvious potential confounding factor (maternal BMI). Moreover, confounding by a general effect of healthy dietary patterns would not give rise to the varying associations with particular micro- and macronutrients that we observed. Further studies in larger numbers of subjects will be required to know if the associations found are truly the effects of the individual nutrients or whether they reflect intakes of the foods rich in these nutrients. Metagenomic sequencing or personalized culture collections could provide further indications of strain-specific effects associated with intakes of particular nutrients. For the associations with fat-soluble vitamins, further work is needed to determine whether contaminants in dietary sources rich in vitamin D may play a role.

## Conclusions

Our analyses provide novel inferences linking the mother’s dietary intake of specific nutrients during pregnancy with her gut microbiota composition in the immediate postpartum period. Given the relative stability of the adult gut microbiome, dietary intervention may be the most effective way to modify intestinal health.

Importantly, the composition of the microbiota in pregnant women at delivery has a direct impact on the gut microbiome of the newborn as maternal microbes initiate the colonization process in the infant. Bäckhed et al. (2015) demonstrated shared microbial features in the mother and infant gut microbiome, including several important microbial species such as *Bifidobacterium longum* and *Bacteroides fragilis* [34], pointing to transfer of microbes from maternal gut microbiota to the child. A recently published study on Sprague Dawley rats demonstrated the effects of maternal gut microbiota and metabolomic profiles on modulating the risk of obesity [35], which indicates the implication on infant health outcomes at least in animal studies. Thus, the observed compositional shifts in maternal gut microbiota may lead to corresponding shifts in infant gut microbiota and affect long-term infant health [36]. Future studies are needed where dietary intakes of fat and fat soluble vitamins during pregnancy are studied in further detail in context of the early infant microbiota and may provide helpful insights to optimize dietary recommendations during pregnancy.

## Methods

### Description of the cohort

NoMIC is a birth cohort established for the overall purpose of studying the establishment of the gut microbiota during infancy and its consequences for child health [37]. Participating mothers were recruited to NoMIC by a pediatrician at the maternity ward in a county hospital in South-Norway. When a preterm-birth mother was enrolled, two mothers of consecutively born term infants were recruited. Hence, the NoMIC cohort has an oversampling of preterm deliveries and is not a proper representative cohort for the general Norwegian population. Recruitment started in November 2002 and was completed in May 2005. Mothers who were fluent in Norwegian were eligible for the cohort.

Containers for fecal samples and a questionnaire were handed out to the participants at the maternity ward after informed consent form had been given. Mothers were asked to collect and freeze one fecal sample from herself at postpartum day 4, as well as samples from her infant when it was 4, 10, 30, 120 days and 1 and 2 years old. Study personnel retrieved samples and kept them frozen during transport. Samples were stored at −20 °C at the Biobank of the Norwegian Institute of Public Health upon arrival. More details of the study population have been given previously [37, 38]. Linkage to the Norwegian Medical Birth Registry (MBR) [39] and linkage to the MoBa study were performed for the study subjects. This provides prospective data on exposures during pregnancy as well as the opportunity to quality check maternal reports.

In total, 608 mothers agreed to participate in the study and to collect fecal samples at six time points during the first 2 years of age. Sixty mothers (9.9 %) did not return any samples. Microbial data was available for 183 mothers and 60 of these 183 women also had dietary information collected through linkage to the MoBa study. Maternal gut microbiota sequenced from these 60 mothers represents the study sample in the current paper.

### Exposure—dietary and other variables

Dietary intakes were recorded through an extensive food frequency questionnaires (FFQ) administered to MoBa participants in week 22 of pregnancy, covering 255 food items through 40 groups of questions (available at https://www.fhi.no/globalassets/migrering/dokumenter/pdf/instrument-documentation-q2.pdf). Food frequencies were converted to nutrient intakes using FoodCalc (http://www.ibt.ku.dk/jesper/foodcalc) and the Norwegian Food Composition Table [40]. The nutrient intake data has been validated in previous publications [33]. The validation studies concluded that the MoBa FFQ is a valid tool that is equipped to measure nutrient intake in pregnant women and can distinguish between women with high and low intake. Intakes of 34 dietary components including energy, macro-, and micronutrients, and water were available. Vitamins and minerals were measured in milligrams or micro-grams while fat, carbohydrates, and protein were measured in grams. Information on intake of multi-vitamin supplements including cod liver oil and omega-3 fatty acids were obtained from separate MoBa questionnaires administered at 15 and 30 weeks and used for sensitivity analysis.

### Sequencing and data processing

Sequencing of the V4 region of the 16S rRNA gene using the Illumina HiSeq instrument resulted in a total of 183 maternal samples with an average of 108,743 (+/−68,090) sequences per sample after demultiplexing. Unless noted otherwise, all data processing was performed using QIIME 1.7.0 [41]. A closed-reference OTU picking procedure was used to map sequences against the Greengenes 13-8 reference set of 16S rRNA gene sequences. Information on DNA purification and PCR is provided in Additional file 1.

### Statistical analysis

For descriptive purposes, we studied the ranges of the dietary components and correlations between the standardized dietary variables (subtracting the mean and dividing by the standard error). Pearson’s correlation was used to study the correlation between these dietary components. The QIIME [41] pipeline was used to compute measures of alpha and beta diversity, which measure diversity within and between microbial communities, respectively. Alpha diversity was measured using two metrics, Shannon’s diversity and whole tree phylogenetic diversity [42]. A Bayesian model selection procedure [43] was used to identify dietary variables most strongly associated with maternal GM alpha diversity. For each of the top five selected variables, we used standard linear regressions (after adjusting for maternal pre-pregnancy BMI and parity) of diversity on continuous standardized dietary variables and computed the percentage changes in microbial alpha diversity against dietary intake. We analyzed the maternal microbial samples to describe beta-diversity through weighted and unweighted UniFrac [44] measures and principal coordinate analysis of the dissimilarity matrix.

Due to the compositional nature of microbial data, there are intrinsic constraints that impede the use of methodology developed for data in Euclidean space. Also, it is important to note that actual abundances of a particular taxon in an ecosystem (gut) are unobservable and not equal to abundances in the sample (fecal). However, the relative abundance of the taxon in the sample is representative of the same in the gut. Hence, using the principles of ANCOM [20], which is based on Aitchison’s logistic-normal formulation for compositional data [45], we investigated the effects of dietary components on log-ratios of four major microbial phyla (*Actinobacteria*, *Firmicutes*, *Proteobacteria*, and *Bacteroidetes*) and all remaining phyla combined together (denoted by *Other*), with respect to each other, amounting to ten microbial log-ratios as the response variables. We regressed each of these log-ratios on the various dietary variables in a multiple regression model. Thus, there are ten linear models and each model has 28 dietary variables, as listed in Table 2. Since the daily consumption of these 28 dietary variables are different by several orders of magnitude, for each person, we standardized the consumption of each dietary variable across samples by subtracting each value by its mean over all samples and dividing by its standard deviation. Such centered multiple regression analysis is commonly conducted in statistics literature when dealing with situations such as the present situation where the explanatory variables differ by several orders of magnitude to avoid issues related to multicollinearity as well as practical interpretability of the data (see page 155, [46]). For each significant association between a dietary variable and a microbial ratio (Phyla 2/Phyla 1), we compute a fold increase (=exp(*β*), where *β* is the regression coefficient) representing the relative increase in Phyla 2 compared to Phyla 1 for unit increase in the standardized dietary variable. Due to small number of hypothesis (10 in this case), we did not perform multiple testing correction for this particular analysis. For each model, we also provided the multiple *R* ^2^ values that show the proportion of explained variation due to the predictors in the model.

In addition, we also performed a compositional analysis on the data summarized at the genera level, using the combinations of ratio-wise *p* values (according to ANCOM) to detect differentially abundant genera against particular dietary variables used in the above analysis. These analyses were restricted to microbial genera detected in at least 5 % of the total number of subjects in the study. A multiple correction using Benjamini-Hochberg procedure is applied in this analysis controlling for false discoveries at 5 % level. For illustrative purposes, we plotted the log OTU abundances for each detected genus against specific dietary nutrients, categorized as intake below and above the median.

To assess the robustness of the results, we carried out sensitivity analyses for detecting compositional shifts under two different situations. Firstly, we repeated the analysis after adjusting for maternal pre-pregnancy BMI. The second sensitivity analysis was performed on the group of women who did not use vitamin supplements during the month leading into the third trimester.

## Additional file

Additional file 1: A word document containing supplementary information including details of DNA processing, statistical methods, R-code, supplementary tables and figures cited in the article. (DOC 331 kb)

## Abbreviations

BMI: Body mass index
FFQ: Food frequency questionnaire
GM: Gut microbiota
NoMIC: Norwegian microflora study
