# Supplementary material for: Fat and vitamin intakes during pregnancy have stronger relations with a pro-inflammatory maternal microbiota than does carbohydrate intake
Source: Microbiome. 2016 Oct 19;4:55. doi: 10.1186/s40168-016-0200-3 (PMC5070355; doi:10.1186/s40168-016-0200-3)
Supplement: Additional file 1: — A word document containing supplementary information including details of DNA processing, statistical methods, R-code, supplementary tables and figures cited in the article. (DOC 331 kb) [file 40168_2016_200_MOESM1_ESM.doc]

**Supplementary Material**

**Fat and vitamin intakes during pregnancy have stronger relations with a pro-inflammatory maternal microbiota than does carbohydrate intake**

**Author list:** Siddhartha Mandal^1,2^, Keith M Godfrey^3^, Daniel McDonald^4^, Will V. Treuren^5^, Jørgen V. Bjørnholt^1,6,7^, Tore Midvedt^8^, Birgitte Moen^9^, Knut Rudi^10^, Rob Knight^4,11^, Anne Lise Brantsæter^1^, Shyamal D. Peddada^12^, Merete Eggesbø^1*^.

**Affiliation:**

^1^ Department of Environmental Exposure and Epidemiology, Norwegian Institute of Public Health, Oslo, Norway.

^2^ Present address: Public Health Foundation of India, Gurgaon, India

^3^ MRC Lifecourse Epidemiology Unit and NIHR Southampton Biomedical Research Centre, University of Southampton and University Hospital Southampton NHS Foundation Trust

^4^ Department of Pediatrics at the University of California San Diego.

^5^ Department of Microbiology and Immunology, Stanford University, California, USA

^6^ Microbiological department Oslo University hospital, Norway

^7^ Institute for Clinical Medicine, University of Oslo, Norway.

^8^Department of Microbiology, Tumor and Cell biology (MTC), Karolinska Institute, Stockholm, Sweden

^9^ The Norwegian Institute of Food Fisheries and Aquaculture, Aas, Norway

^10^Department of Chemistry, Biotechnology and Food Science, Norwegian Institute of Life Sciences, Aas, Norway

^4,11^ Department of Computer Science, UC San Diego, USA

^12^ Biostatistics and Computational Biology Branch, National Institute for Environmental Health Sciences, NC, USA

***Automated DNA purification of faecal samples:*** Feces were prepared for analysis by adding one milliliter Solution 1 (50 mM glucose, 25 mM Tris–HCl pH 8.0, 10 mM EDTA pH 8.0) per 0.2 g faeces. The samples were mixed by vortexing and left for 30-60 min on ice before 400 µl of the supernatant was diluted 1:2 in 4 M guanidinium thiocyanate (GTC). Five hundred microliters of sample were transferred to a sterile FastPrep®-tube (Qbiogene Inc., Carlsbad, CA, USA) containing 250 mg glass beads (106 microns and finer, Sigma-Aldrich, Steinheim, Germany), and samples were homogenised for 40 seconds in FastPrep® Instrument (Qbiogene). Wells in a 96-well Greiner U-plate (Greiner bio-one, Frickenhausen, Germany) were filled with 170 μl sample and 10 μl Silica particles (Merck, Darmstadt, Germany) and transferred to a Biomek® 2000 Workstation (Beckman Coulter, Fullerton, CA, USA). One percent Sarkosyl was added, and the plate was incubated at 65 °C for 10 min and at room temperature for 10 min. The supernatant was removed, and the paramagnetic beads were washed twice with 50% ethanol. DNA was eluted from the silica particles by suspension of the particles in 100 μl Buffer C (1 mM EDTA pH 8.0, 10 mM Tris–HCl pH 8.0) at 65 °C for 30 min. The adequacy of the automated DNA extraction procedure was evaluated by repeating the DNA extraction in 20 samples using the modified MoBio 96-well manual extraction method adopted by the Earth Microbiome Project (<http://press.igsb.anl.gov/earthmicrobiome/emp-standard-protocols/dna-extraction-protocol/>). The samples gave very similar results regardless of DNA extraction method used.

***PCR:*** 1 ul DNA extracted from faecal samples was amplified by PCR reactions by 16S specific primers (515F-806R). All reactions were set up as 25ul samples in 96 well Thermo-fast 96, low profile, 0,2ml, non skirted PCR plates (ABgene Thermo scientific, UK) with Cas1200 Corbett robot (Qiagen). 10ul HotMastermix enzyme (5PRIME GmbH, Germany), 0.2uM forward-/ reverse primers (ILHS_515fa/ IL_806rcbc) and 13ul PCR grade water (Qiagen) were used.

***Statistical models used for log-ratio analysis:*** We use the main ideas underlying ANCOM (Mandal et al. 2015) for the log-ratio analysis. Consider a microbial composition consisting of *k* taxa (Taxa_1_, … , Taxa_k_) observed on *n* individuals, resulting in relative abundance vectors ***Y****_i_ = (Y_i1_, … , Y_ik_)'* for the *i*-th individual. Note that relative abundance vectors sum to unity within a sample from an individual. Hence **Y**_i_ does not belong to the Euclidean space which is necessary for application of standard statistical methodology. Further the mathematical constraints lead to spurious correlation structure between taxa.

The key point to note is that changes in abundance of one taxon induces changes in the relative abundances of all taxa. So changes in composition are functions of all taxa and not one at a time. We use this fact to analyze shifts in microbial composition against dietary variables. Note that our null hypothesis for this analysis **H_0r_ : No shift in composition** does not make inferences on individual taxa. Further **H_0r_** can be expressed as a combination of ratio-wise hypothesis given by **H_0jj'_: No change in *E[log(Y_j_/ Y_j'_)]***, ***j*≠*j'***. Detailed mathematical results are provided in Mandal et al. 2015.

Towards this we construct log-ratios of pairs of taxa, which results in *k(k-1)/2* ratios. To reduce the number of necessary comparisons, we restrict this analysis to 5 microbial phyla (Actinobacteria, Proteobacteria, Bacteroidetes, Firmicutes and Others), where Others constitute all other phyla. Thus, in this case, the number of possible log-ratios is 10.

Consider the regression of the ratio of any two microbial taxa (Taxa 1 and Taxa 2) on p dietary variables *V_1_*,...,*V_p_*, given by: *log(Y_j_/ Y_j'_) = β_0_ + β_1_ V_1_ + … + β_p_ V_p_* . To counter the problem of zeroes in the taxa relative abundances, we add 0.001 to the taxa relative abundances, while computing the ratios. The coefficient β_j_ denote the change in the log-ratio for unit standard deviation increase in V_j_. The fold change in Taxa *j* : Taxa *j'*, corresponding to *V_j_* is computed as exp(β_j_). For example, in the analysis of associations against fat-soluble vitamins, *V_1_*, *V_2_* and *V_3_* are Vitamin D, Vitamin E and Retinol while the taxa are the 5 microbial phyla mentioned earlier. The p-values obtained from the log-ratio analysis (denoted by *p_jj'_,* *j*≠*j'*) corresponding to taxa *j*, are dependent since *Y_j_ / Y_j'_* (for fixed *j*) are dependent random variables. We do not use multiple correction for this particular analysis since the number of pre-specified dependent hypothesis are small (10 in this case).

For the second part of the analysis involving genus level data, the hypothesis of interest is **H_0gj_: Genus *j* is not differentially abundant across dietary levels**. This requires the combination of the ratio-wise p-values obtained from the log-ratio analysis. We construct microbial ratios based on genera level relative abundances. Following ANCOM, we impose a multiple correction using Benjamini-Hochberg procedure at 5% level of significance within all pairwise hypothesis for each taxon. Let *q_jj'_*, *j*≠*j'*, *j,j' =1,...,k* be the p-values corresponding to the *j*-th taxa (after multiple correction using BH procedure) and *W_j_* be the number of *q_jj'_* less than 0.05. A high value of *W_j_* indicates the rejection of **H_0jj'_** for large number of *j'*(≠*j*). Thus the decision rule to reject **H_0gj_** is given by *W_j_ /(k-1) > 0.75*.

***Implementation:*** R codes for the multiple regression results involve standard implementations using the lm() function. Implementation of the code is provided below. A detailed code for ANCOM analysis at genus level can also be obtained from the following webpage: [*http://www.niehs.nih.gov/research/atniehs/labs/bb/staff/peddada/*](http://www.niehs.nih.gov/research/atniehs/labs/bb/staff/peddada/).

### Creating the ratio data from the original data (denoted by ancom.mom) which has the sample ids and phyla relative abundances in each column with ### each row denoting a microbial sample.

taxa.names=c("Actinobacteria","Firmicutes","Proteobacteria","Bacteroidetes","Others")

ratio.names=as.character(taxa.names)

ratio.comb=combn(ratio.names, 2)

ratio.data.phyla=ancom.mom[,1:2]

for(i in 1:dim(ratio.comb)[2]){

taxa1=ancom.mom[,which(colnames(ancom.mom)==ratio.comb[1,i])]

taxa2=ancom.mom[,which(colnames(ancom.mom)==ratio.comb[2,i])]

#outcome=scale(log((0.001+taxa1)/(0.001+taxa2)),center=T,scale=T)

outcome=log((0.001+taxa1)/(0.001+taxa2))

ratio.data.phyla=data.frame(ratio.data.phyla,outcome,row.names=NULL)

}

colnames(ratio.data.phyla)=c("kortnr","Sample.ID",paste0("Phyla.R",c(1:10)))

ratio.data=data.frame(ratio.data.phyla,ancom.mom[,8:42],row.names=NULL)

y=data.matrix(ratio.data[,1:12])

x=scale(data.matrix(ratio.data[,13:47]),center=TRUE,scale=TRUE)

colnames(y)=c("kortnr","Sample.ID",apply(ratio.comb,2,function(x){

s=paste0(substr(x[1],1,1),"/",substr(x[2],1,1))

return(s)

}))

full.data=data.frame(y,x,row.names=NULL)

### Full multiple linear regression model for microbial ratios against dietary variables.

full.model=lm(cbind(A.F,A.P,A.B,A.O,F.P,F.B,F.O,P.B,P.O,B.O)~Protein.Total+

Fat.Saturated+Fat.Total.trans+Fat.Monounsaturated+Fat.Polyunsaturated+Cholesterol+

Carb.Starch+Carb.Fiber+Glucose.sachharide+Glucose.Sugar+Vit.Retinol+Vit.Betacarotene+

Vit.D+Vit.E+Vit.Thiamine+Vit.Riboflavin+Vit.Niacin+Vit.B6+Vit.Folate+Vit.B12+Vit.C+

Min.Potassium+Min.Magnesium+Min.Zinc+Min.Selenium+Min.Copper+Min.Phosphorus,data=full.data)

### Function to detect differentially abundant genera according to dietary variable

ancom.detect=function(otu_data,n_otu,alpha,multcorr){

logratio.mat=matrix(NA,nr=n_otu,nc=n_otu)

for(i in 1:(n_otu-1)){

for(j in (i+1):n_otu){

data.pair=otu_data[,c(i,j,n_otu+1)]

lr=log((0.001+as.numeric(data.pair[,1]))/(0.001+as.numeric(data.pair[,2])))

logratio.mat[i,j]=wilcox.test(lr[data.pair$grp==unique(data.pair$grp)[1]],lr[data.pair$grp==unique(data.pair$grp)[2]])$p.value

}

}

ind <- lower.tri(logratio.mat)

logratio.mat[ind] <- t(logratio.mat)[ind]

logratio.mat[which(is.finite(logratio.mat)==FALSE)]=1

mc.pval=t(apply(logratio.mat,1,function(x){

s=p.adjust(x, method = "BH")

return(s)

}))

a=logratio.mat[upper.tri(logratio.mat,diag=F)==T]

b=matrix(0,nc=n_otu,nr=n_otu)

b[upper.tri(b)==T]=p.adjust(a, method = "BH")

diag(b)=NA

ind.1 <- lower.tri(b)

b[ind.1] <- t(b)[ind.1]

# W.original=apply(logratio.mat,1,function(x){

# subp=length(which(x<0.05))

# })

if(multcorr==T){

W=apply(mc.pval,1,function(x){

subp=length(which(x<alpha))

})

}else if(multcorr==F){

W=apply(logratio.mat,1,function(x){

subp=length(which(x<alpha))

})}

return(W)

}

### Data to use for detecting genera different according to Vitamin D

diet_data=ancom.mom[,c("Sample.ID","Vit.D")]

diet_data$Vit.D=cut(diet_data$Vit.D,

c(0,quantile(diet_data$Vit.D,0.5),

1+max(diet_data$Vit.D)),labels=c(1,2))

diet_data=merge(otu_mandc_genus,diet_data,by="Sample.ID",all.y=T)

data.1=diet_data

real.data = data.1[,-1]

colnames(real.data)[dim(real.data)[2]]="grp"

real.data = data.frame(real.data[which(is.na(real.data$grp)==F),],row.names=NULL)

par1_new=dim(real.data)[2]-1

W.detected=ancom.detect(real.data,par1_new,0.05,T)

W.detected

colnames(real.data)[which(W.detected>407*0.75)]

if(max(W.detected)/par1_new >=0.10){

c.start=max(W.detected)/par1_new

cutoff=c.start-c(0.05,0.10,0.15,0.20,0.25)

prop_cut=rep(0,length(cutoff))

for(cut in 1:length(cutoff)){

prop_cut[cut]=length(which(W.detected>=par1_new*cutoff[cut]))/length(W.detected)

}

del=rep(0,length(cutoff)-1)

for(i in 1:(length(cutoff)-1)){

del[i]=abs(prop_cut[i]-prop_cut[i+1])

}

if(del[1]<0.02&del[2]<0.02&del[3]<0.02){nu=cutoff[1]

}else if(del[1]>=0.02&del[2]<0.02&del[3]<0.02){nu=cutoff[2]

}else if(del[2]>=0.02&del[3]<0.02&del[4]<0.02){nu=cutoff[3]

}else{nu=cutoff[4]}

up_point=min(W.detected[which(W.detected>=nu*par1_new)])

W.detected[W.detected>=up_point]=99999

W.detected[W.detected<up_point]=0

W.detected[W.detected==99999]=1

}else{W.detected=0}

colnames(real.data)[which(W.detected==1)]

***Supplementary Figures:***

*Figure S1: Variables selected by bayesian model averaging arranged according to decreasing posterior model inclusion probability for whole tree phylogenetic diversity (left panel) and Shannon's diversity (right panel). Vertical dotted lines represent the selected models and the colors denote negative (red) and positive (blue) associations between alpha diversity and corresponding dietary nutrient. For example, in the left panel, Vitamin D is included in the two best models (with cumulative posterior inclusion probability of 0.30 and 0.35) and it is consistently negatively associated with phylogenetic diversity. The other variables do not feature consistently in the top models.*


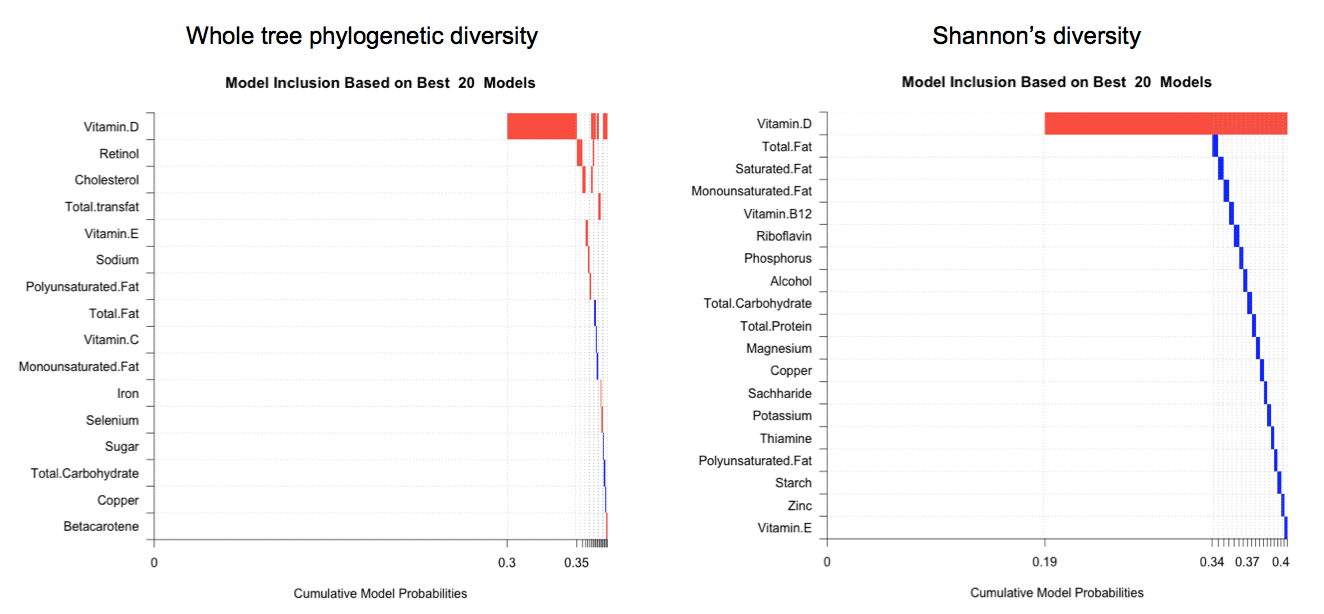


***Supplementary Tables:***

*Table S1: Percentage change in (a) whole tree phylogenetic diversity and (b) Shannon's diversity, against top 5 dietary variables selected by bayesian variable selection, after adjusting for maternal pre-pregnancy BMI and parity. Percentage change is measured by 100*b_1_/b_0_, where b_0_ and b_1_ are the regression coefficients for intercept and the dietary variable in a linear regression.*

(a)

(b)

*Table S2:* Estimates of change in log ratios of microbial phyla against dietary variables in 60 mothers, according to a multiple linear regression model. For example, there is a exp(1.289) = 3.63 times increase in Actinobacteria compared to Proteobacteria for 1 SD increase in Vitamin E intake. Corresponding p-values and R squared values for each model is provided in the table.

*Table S3:* Estimates of change in log ratios of microbial phyla against dietary variables in mothers, according to a multiple linear regression model adjusted for maternal pre-pregnancy BMI and gestational age (in days). For example, there is a exp(1.521) = 4.58 times increase in Actinobacteria compared to Proteobacteria for 1 SD increase in Vitamin E intake. Corresponding p-values and R squared values for each model is provided in the table.

*Table S4:* Supplementary Table 4: Estimates of change in log ratios of microbial phyla against dietary variables in 39 mothers who delivered in term, according to a multiple linear regression model. For example, there is a exp(1.289) = 3.75 times increase in Actinobacteria compared to Proteobacteria for 1 SD increase in Vitamin E intake. Corresponding p-values and R squared values for each model is provided in the table.

*Table S5:* Estimates of change in log ratios of microbial phyla against dietary variables in mothers who did not use supplements for Vitamin D, E and Retinol, according to a multiple linear regression model. Corresponding p-values and R squared values for each model is provided in the table.
